# Uncovering salt tolerance mechanisms in pepper plants: a physiological and transcriptomic approach

**DOI:** 10.1186/s12870-021-02938-2

**Published:** 2021-04-08

**Authors:** Lidia López-Serrano, Ángeles Calatayud, Salvador López-Galarza, Ramón Serrano, Eduardo Bueso

**Affiliations:** 1 Centro de Citricultura y Producción Vegetal, Departamento de Horticultura, Instituto Valenciano de Investigaciones Agrarias, CV-315, Km 10,700 Moncada, Valencia, Spain; 2 Departamento de Producción Vegetal, Universitat Politècnica de València, Valencia, Spain; 3 Instituto de Biología Molecular y Celular de Plantas, Universidad Politécnica de Valencia-C.S.I.C, Camino de Vera s/n, 46022 Valencia, Spain

**Keywords:** Abscisic acid, Growth, Ion homeostasis, Photosynthesis, Salt stress, Tolerant accessions, Pepper

## Abstract

**Background:**

Pepper is one of the most cultivated crops worldwide, but is sensitive to salinity. This sensitivity is dependent on varieties and our knowledge about how they can face such stress is limited, mainly according to a molecular point of view. This is the main reason why we decided to develop this transcriptomic analysis. Tolerant and sensitive accessions, respectively called A25 and A6, were grown for 14 days under control conditions and irrigated with 70 mM of NaCl. Biomass, different physiological parameters and differentially expressed genes were analysed to give response to differential salinity mechanisms between both accessions.

**Results:**

The genetic changes found between the accessions under both control and stress conditions could explain the physiological behaviour in A25 by the decrease of osmotic potential that could be due mainly to an increase in potassium and proline accumulation, improved growth (e.g. expansins), more efficient starch accumulation (e.g. BAM1), ion homeostasis (e.g. CBL9, HAI3, BASS1), photosynthetic protection (e.g. FIB1A, TIL, JAR1) and antioxidant activity (e.g. PSDS3, SnRK2.10). In addition, misregulation of ABA signalling (e.g. HAB1, ERD4, HAI3) and other stress signalling genes (e.g. JAR1) would appear crucial to explain the different sensitivity to NaCl in both accessions.

**Conclusions:**

After analysing the physiological behaviour and transcriptomic results, we have concluded that A25 accession utilizes different strategies to cope better salt stress, being ABA-signalling a pivotal point of regulation. However, other strategies, such as the decrease in osmotic potential to preserve water status in leaves seem to be important to explain the defence response to salinity in pepper A25 plants.

**Supplementary Information:**

The online version contains supplementary material available at 10.1186/s12870-021-02938-2.

## Background

Pepper (*Capsicum annuum* L.) is one of the most important cultivated horticultural species worldwide. Production has increased over the last 20 years from 17 to 36 million tons, and the cultivated area has expanded by about 35% [1]. However, several stresses still significantly affect peppers, which decrease yields and fruit quality. The most important stress is biotic, but peppers are also affected by some abiotic stresses [2]. One of the most relevant ones is salt stress as pepper plants are considered moderately sensitive, sensitive or highly susceptible [3, 4]. The source of a high salt concentration that affects plants may be either soil or irrigation water [5]. In pepper plants, dry weight and marketable yield diminished by 46 and 25%, respectively when is irrigated with water at 4.4 dS m^− 1^ [6].

The root is the first organ affected after the exposure to high Na^+^ and Cl^−^ concentration, since the excess of these ions generates osmotic and ionic stress [4, 7]. These ions also move rapidly to photosynthetic organs and cause several negative effects. Indeed salt accumulation in plant tissues provokes changes in the physiological metabolism, such as nutritional imbalances, and generates reactive oxygen species (ROS), among other physiological disorders that lead to reduce biomass and crop production [8, 9]. However, some species are able to deal with these negative effects and can be tolerant to salt stress. To reach this condition, a complex network of genes related to salt tolerance is necessary [10], that can modify physiological and biochemical plant responses.

In agricultural species, growers have always tended to select genotypes with increased commercial production, commonly linked to improved tolerance to specific stresses. As a result, it is now possible to find a wide diversity of accessions that differs in terms of grades of tolerance to stresses. In the case of pepper, several authors have demonstrated that the severity of negative effects depends on the variety [11–14].

This intraspecies variation may be a source of information to find factors like genes, proteins or metabolites related to tolerance, which can be used in, for example, conventional breeding programmes or genetic engineering technologies [10], or to be employed as tolerant rootstocks in grafted plants [15, 16].

Several transcriptomic studies revealed the understanding of the genetic mechanisms responsible for the tolerance of pepper plants to various stresses, such as heat stress, chilling or leaf curl virus [17–20]. In the case of salt stress, specific genetic pathways of tolerance has been addressed [21–23] but scarce information is available related to pepper plants.

Consequently, this study compared two pepper accessions previously classified by us as tolerant (A25) and sensitive (A6) to salt stress after analysing a series of physiological and agronomical parameters [12, 24]. This study included a series of measurements to evaluate different physiological traits, as well as a transcriptomic analysis, by microarrays, to elucidate the genetic programmes that were expressed and are responsible for tolerance to salt stress. This analysis could reveal the underlying mechanisms in pepper to cope with salinity stress and open up new strategies to improve crop performance under salinity conditions.

## Results

### Biomass

**Fig. 1:**
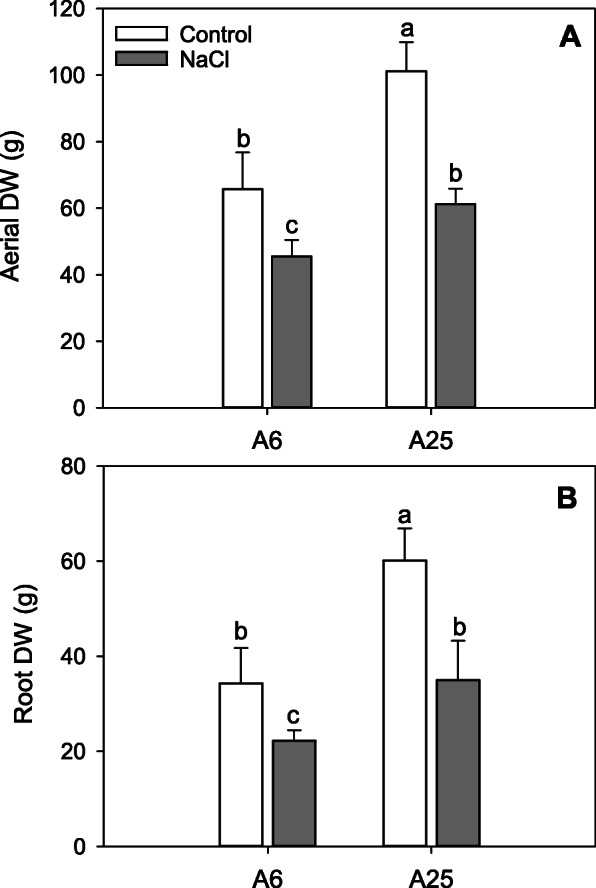
Dry weight of the aerial part (**a**) and the root zone (**b**), in the accessions A6 and A25, under control and salt stress (70 mM NaCl) conditions. Measurements were taken at the end of the experiment (14DAT). Data are the mean of 6 replicates and the error bars belong to the standard deviation. Different letters indicate significant differences at *P* < 0.05 (LSD test)

In order to evaluate whether plants maintained the same growth rate after NaCl treatment, dry biomass was measured in both roots and aerial organs at 14 days after treatment (14DAT). Under the control conditions, both accessions obtained higher values compared to the salt stress conditions (Fig. 1a, b). Nevertheless, growth under the control conditions differed between accessions as A25 obtained higher values in both aerial and root biomass (Fig. 1a, b). The tolerant A25 accession better maintained both aerial and root dry weight under salt stress conditions compared to A6 accession at the end of the experiment (14DAT).

### Gas exchange measurements

**Fig. 2:**
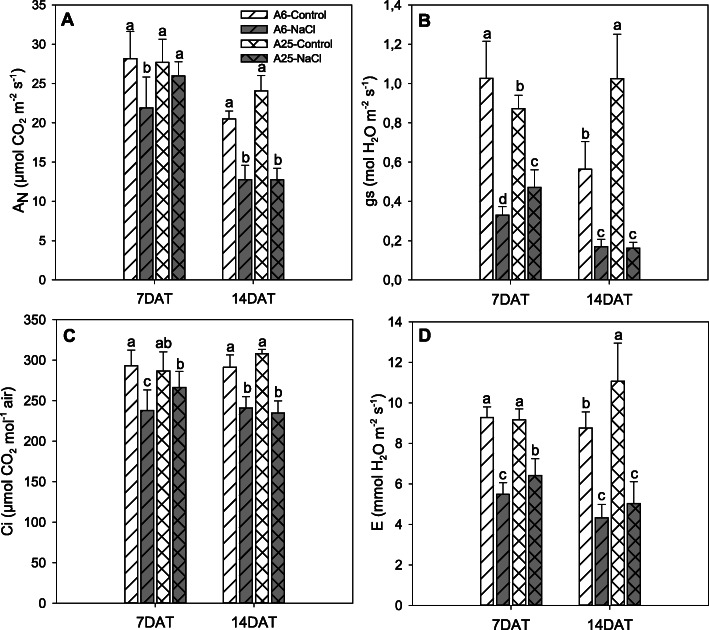
CO_2_ fixation rate (A_N_, μmol CO_2_ m^− 2^ s^− 1^) (**a**), stomatal conductance to water vapour (gs, mol H_2_O m^− 2^ s^− 1^) (**b**), substomatal CO_2_ concentration (Ci, μmol CO_2_ mol ^− 1^ air) (**c**) and transpiration rate (E, mmol H_2_O m^− 2^ s^− 1^) (**d**) under control and salt stress (70 mM NaCl) conditions. Measurements were taken after 7 days (7DAT) and 14 days (14DAT) of the experiment. Data are the mean of 5 replicates and the error bars belong to the standard deviation. For each studied time, different letters indicate significant differences at *P* < 0.05 (LSD test)

As photosynthesis is one of the first processes affected after salt stress exposition, so it is crucial to evaluate its parameters and how they progress with time exposure. In this experiment, CO_2_ assimilation rate (A_N_), stomatal conductance to water vapour (gs), substomatal CO_2_ concentration (Ci) and transpiration rate (E) were analysed at 7DAT and 14DAT (Fig. 2). At 7DAT, A25 showed no significant differences in A_N_ and Ci (Fig. 2a, c) between the control and salt stress conditions. Conversely, gs and E decreased in the stressed plants (Fig. 2b, d) but, compared to A6, these parameters in A25 were better maintained as A6 obtained the lowest values of them all.

At 14DAT under the control conditions, a better response was observed in stomatal conductance and transpiration with the tolerant accession A25 (Fig. 2b, d), unlike A_N_ and Ci, which remained unchanged (Fig. 2a, c). Different results were found under salt stress conditions, with no significant differences between the two studied accessions for all the gas exchange parameters studied, that scored similar values.

### Ion determination

**Fig. 3:**
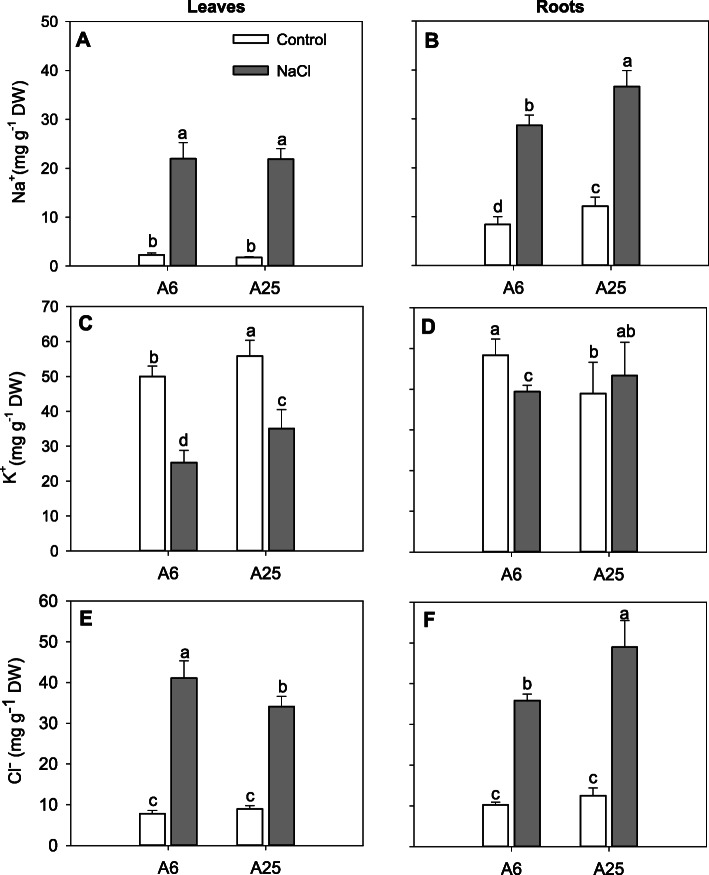
Na^+^ (**a, b**), K^+^ (**c, d**) and Cl^−^ concentration (**e, f**) in leaves (**A, c, e**) and roots (**b, d, f**) in the accessions A6 and A25 under control and salt stress (70 mM) conditions. Measurements were taken at the end of the experiment (14DAT). Data are the mean of 6 replicates and the error bars belong to the standard deviation. Different letters indicate significant differences at *P* < 0.05 (LSD test)

Exposure to high NaCl concentrations disrupts ion homeostasis in plant cells [8]. Thus, the evaluation of the ion concentration in different tissues after exposure to stress was crucial for this experiment. For this purpose, Na^+^, K^+^ and Cl^−^ concentrations were measured at the end of the experiment (14DAT) in leaves (Fig. 3a, c, e) and roots (Fig. 3b, d, f). Regarding Na^+^ (Fig. 3a, b), the concentration in both leaves and roots increased for the two accessions studied when were subjected to salt stress. It is worth mentioning that the levels in roots were higher than leaves for both accessions and treatments, especially in A25, which showed more Na^+^ accumulation compared to A6.

Regarding the K^+^ concentration (Fig. 3c, d), under high salinity treatment the leakage was higher in leaves compared to roots. Nonetheless, the potassium level remained constant in the roots of the A25 accession in both conditions. In addition, K^+^ levels were higher in A25 under salt stress in both leaves and roots compared to A6.

Besides, a higher Cl^−^ concentration was detected under the salt conditions in all the studied organs and accessions compared to the control (Fig. 3e, f). However, the concentration in the A25 accession under salt stress rose in roots and lowered in leaves compared to A6. Under the control conditions, no significant differences were found in any of the studied organs.

### Physiological determinations

**Fig. 4:**
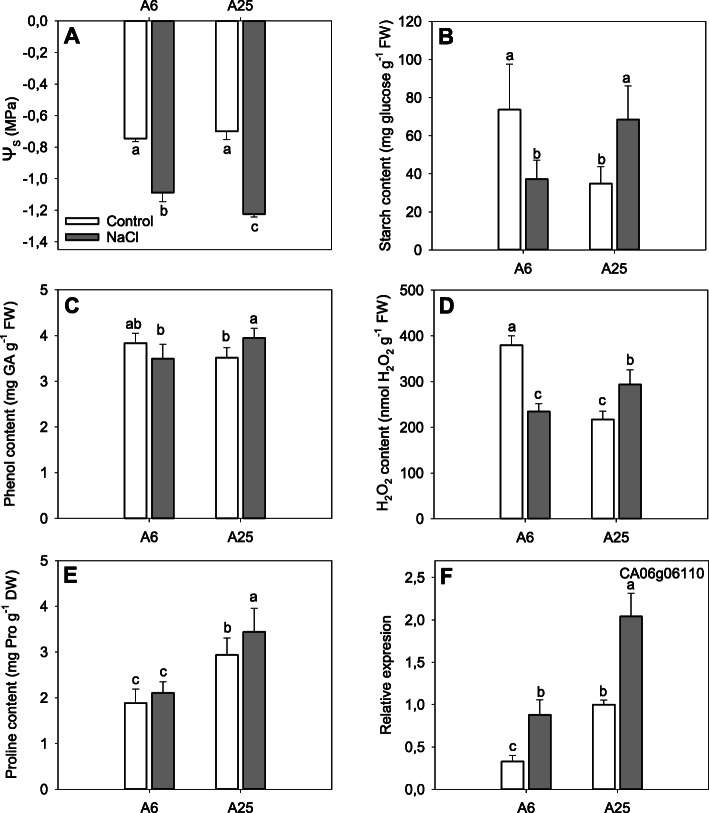
Osmotic potential (**a**), starch content (**b**), total phenol content (**c**), H_2_O_2_ content (**d**), proline concentration (**e**) and *CaP5CS* (CA06g06110) gene relative expression (**f**) in the leaves of the accessions A6 and A25 under control and salt stress (70 mM) conditions. Measurements were taken at the end of the experiment (14DAT). Data are the mean of 4 replicates, except for proline content, which is the mean of 6 replicates. The error bars belong to the standard deviation. Different letters indicate significant differences at *P* < 0.05 (LSD test)

The evaluation of the osmotic potential (ψ_S_), the starch content as well as the phenols, proline and H_2_O_2_ concentration can additionally inform about the capacity of plants to tolerate or not salt stress conditions (Fig. 4).

The ψ_S_ evaluated in the leaves at 14DAT (Fig. 4a) showed that, unlike control conditions, salt stress conditions displayed significant differences between the tolerant and the sensitive accessions, where A25 reached the lowest values.

After starch content analysis we could that the accessions present very different behaviours in both control and stress conditions (Fig. 4b). Concretely, starch content decreased in A6, at 14DAT, in the case of salt stress conditions, lowering to the values of control conditions of A25. On the contrary, A25 had significant higher values in the case of salt stress when compared to its control or A6 under salt stress conditions.

In the case of the total phenol concentration in the leaves (Fig. 4c), we did not find significant differences in the case of control conditions among both accessions at the end of the experiment. However, after salt stress, significant differences were observed, reaching the highest values in the case of A25.

Concerning H_2_O_2_ determination (Fig. 4d), differential accumulation in leaves was detected among the tolerant and the sensitive accessions at 14DAT. In the case of control conditions, A6 reached the highest accumulation, decreasing when salt stress was present. On the contrary, A25 significantly increased H_2_O_2_ levels if compared to its control conditions or A6 accession under salt stress.

Finally, proline content and relative expression of its putative gene CaP5CS (CA06g06110) in leaves have been determined in the end of the experiment (Fig. 4e-f). In the case of proline content (Fig. 4e), A6 reported the lowest values, without significant differences between control and salt stress conditions; on the contrary, A25 reported more accumulation under salt stress than control conditions or the A6 accession. Similar results were found in the case of the relative expression of the gene CaP5CS (Fig. 4f), whose expression in A25 under salt stress conditions significantly increased 2-fold respect to its control or A6 under salt stress; nevertheless, we have detected as well an increase of CaP5CS expression in the case of A6 under salt stress conditions compared to its control.

### Transcriptomic expression results

A microarray experiment analysis was performed to know the transcriptomic changes that could explain the sodium chloride tolerance of the A25 accession.

**Fig. 5:**
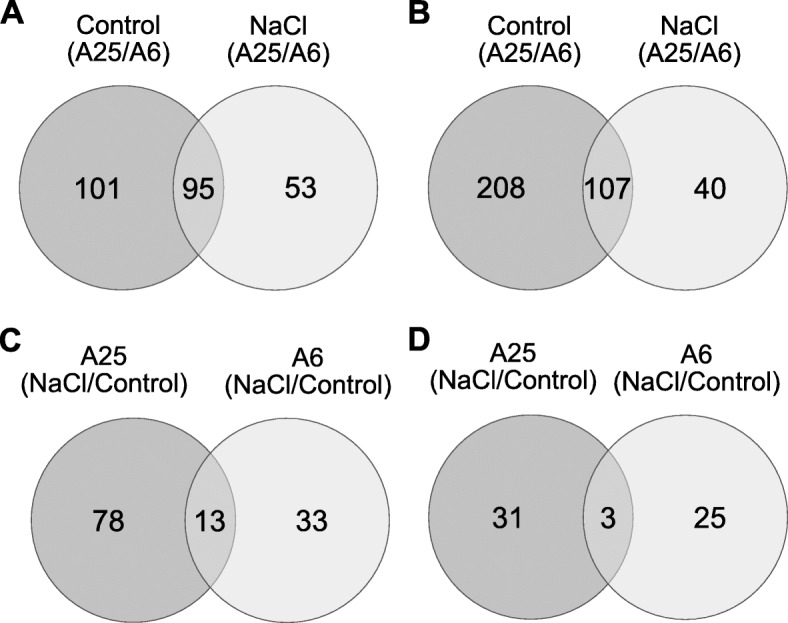
Overlap of the up-regulated (**a, c**) and down-regulated (**b, d**) DEGs between the accessions in the different comparisons at 14DAT. A and B represent Venn diagram analysis of DEGs in A25 respect to A6 in control and salt stress conditions. C and D represent Venn diagram analysis of DEG of each accession when salt stress is compared to control conditions

Under the control conditions when the A25 accession was compared to A6, 196 and 315 genes were up- and down-regulated, respectively (Fig. 5a, b), of which 95 up- and 107 down-regulated genes were commonly expressed in control and salt stress conditions. Of all these genes, it is important to highlight the up-regulated genes related to cell wall biosynthesis and expansion (PMEI13, TUB8, EXPA13, XK-1, PME1, CEL5, CSLE1), wax and fatty acid biosynthesis (KASI, LACS2), cell division (CDC2), vitamin transport (BASS1), ABA-signalling (SnRK2.10, TINY2 and ERD4) and photosynthesis (PSBP-1) in A25 vs A6. The genes related to the formation of cellular barriers, such as lignins (PRX71, PRX66) and waxes (WS6D, CER1), were down-regulated in A25 compared to A6. The down-regulation of the genes involved in stress protection (CAMTA5, JAR1, CBL9) and photosynthesis (NDHG, CCB3) (Tables 1, 2; Additional file 1) was noteworthy. In accordance with these findings, functional GO analysis displayed related results (Additional file 2, Fig. S1).

**Table 1:** Summary of the specific differentially expressed genes after 14DAT in the comparison A25/A6 in plants subjected to control conditions. It is represented both the up (FC > 1) and down-regulated genes (FC < 1), as well as the fold change (FC) and the adjusted *P*-value obtained for each gene (significant differences were considered when *P* < 0.05). Genes without abbreviation are represented with “-“

Full name	Short name	FC	*P*-value	*C. annuum* code	*A. thaliana* code
DNA-directed RNA polymerase subunit beta (Protein of unknown function. DUF642)	–	3.1	8.80E-03	CA01g20890	AT3G08030
Pectin lyase-like superfamily protein	–	2.3	6.00E-03	CA00g70080	AT3G07820
Expansin A13	EXPA13	2.3	0.05	CA04g04060	AT3G03220
Rubisco methyltransferase family protein	–	1.9	9.18E-03	CA08g02430	AT1G24610
Plant invertase/pectin methylesterase inhibitor superfamily protein	PMEI13	1.9	0.04	CA03g15820	AT5G62360
cation/hydrogen exchanger 14	CHX14	1.4	0.02	CA06g25650	AT1G06970
Tubulin beta 8	TUB8	1.4	0.02	CA06g25000	AT5G23860
Peroxidase superfamily protein	–	0.7	0.03	CA00g44710	AT2G37130
GDSL-like Lipase/Acylhydrolase superfamily protein	–	0.7	0.04	CA10g03820	AT5G45960
Eceriferum 1	CER1	0.7	0.02	CA01g27070	AT1G02205
Photosystem I assembly protein	YCF3	0.6	0.01	CA00g81520	ATCG00360
Calcineurin B-like protein 9	CBL9	0.6	0.04	CA01g33680	AT5G47100
O-acyltransferase (WSD1-like) family protein	WSD6	0.6	0.02	CA00g64820	AT3G49210
Cytochrome P450. family 86. subfamily A. polypeptide 8	CYP86A8	0.6	0.04	CA08g07320	AT2G45970
Beta-amylase 5	BAM5	0.6	0.02	CA07g12430	AT4G15210
Rubisco methyltransferase family protein	LSMT-L	0.5	0.01	CA11g04070	AT1G14030
Cellulose synthase family protein	CEV1	0.5	0.01	CA01g20250	AT5G05170
Pectin lyase-like superfamily protein	–	0.5	0.03	CA09g01850	AT3G53190
Jasmonate resistant 1	JAR1	0.4	1.45E-03	CA08g08190	AT2G46370
Calmodulin-binding transcription activator 5	CAMTA5	0.3	1.46E-03	CA01g14110	AT4G16150

**Table 2:** Summary of the common differentially expressed genes after 14DAT in the comparison A25/A6 in plants subjected to control and salt stress conditions. It is represented both the up (FC > 1) and down-regulated genes (FC < 1), as well as the fold change (FC) and the adjusted *P*-value obtained for each gene (significant differences were considered when *P* < 0.05). Genes without abbreviation are represented with “-“

Full name	Short name	Control		NaCl		*C. annuum* code	*A. thaliana* code
		FC	*P*-value	FC	*P*-Value		
Cell division control 2	CDC2	13.6	9.57E-05	6.9	1.15E-03	CA12g18420	AT3G48750
Xylulose kinase-1	XK-1	11.5	6.96E-06	11.0	1.48E-05	CA12g08890	AT2G21370
Sodium Bile acid symporter family	BASS1	7.1	7.34E-03	22.0	2.28E-04	CA09g06260	AT1G78560
SNF1-related protein kinase 2.10	SnRK2.10	3.6	3.20E-04	4.0	1.78E-04	CA08g14400	AT1G60940
Pectin methylesterase 1	PME1	2.5	3.37E-05	2.7	2.15E-05	CA03g36990	AT1G53840
Early-responsive to dehydration stress protein (ERD4)	ERD4	2.4	5.47E-04	2.0	4.87E-03	CA08g02700	AT1G30360
Photosystem II subunit P-1	PSBP-1	2.1	1.34E-04	2.3	4.80E-05	CA07g07930	AT1G06680
3-ketoacyl-acyl carrier protein synthase I	KASI	2.0	0.02	4.2	1.49E-04	CA01g00840	AT5G46290
3-ketoacyl-acyl carrier protein synthase I	KASI	2.0	0.02	4.2	1.49E-04	CA01g00830	AT5G46290
Cellulase 5	CEL5	1.7	3.86E-03	2.0	4.59E-04	CA11g09950	AT1G22880
Integrase-type DNA-binding superfamily protein	TINY2	1.7	3.86E-03	2.0	4.59E-04	CA08g04820	AT5G11590
Long-chain acyl-CoA synthetase 2	LACS2	1.6	3.86E-03	1.9	6.64E-04	CA08g18140	AT1G49430
Cellulose synthase like E1	CSLE1	1.5	0.02	1.5	0.02	CA05g16620	AT1G55850
ERD (early-responsive to dehydration stress) family protein	–	0.7	0.03	0.7	0.03	CA06g26780	AT4G02900
Peroxidase 71	PRX71	0.6	4.03E-04	0.6	2.18E-04	CA12g06550	AT5G64120
Peroxidase 71	PRX71	0.5	1.49E-03	0.7	0.04	CA12g06580	AT5G64120
Eceriferum 1	CER1	0.5	0.03	0.5	0.05	CA01g19130	AT1G02205
Peroxidase 66	PRX66	0.4	1.25E-04	0.6	0.01	CA03g16810	AT5G51890
Xyloglucan endotransglucosylase/hydrolase 7	XTH7	0.3	1.13E-05	0.4	3.30E-05	CA02g24640	AT4G37800
NADH:ubiquinone/plastoquinone oxidoreductase, chain 6	NDHG	0.3	1.05E-03	0.4	9.44E-03	CA08g09370	ATCG01080
Cofactor assembly, complex C (B6F)	CCB3	0.1	6.96E-06	0.1	2.04E-05	CA02g03840	AT5G36120

We could check the abundance of genes related to “response to stress”, “response to abiotic or biotic stimulus”, “transport” and “signal transduction”. Regarding to cellular components we highlight the significative number of genes related to “chloroplast”, “cell wall” or “plastid”. KEGG pathways (Additional file 1, Table S1–2), showed significative results in this set of genes. “Biosynthesis of secondary metabolites”, “Cutin, suberine and wax biosynthesis”, “Fructose and mannose metabolism” and “carbon fixation in photosynthetic organisms” were enriched pathways in downregulated genes.

The response of each accession to salt stress (NaCl/Control) was very different as only 13 up- and 3 down-regulated genes were commonly expressed (e.g. CSD1, MIOX1) (Fig. 5c, d). In relation to A25 accession 78 and 31 genes were specifically expressed (Fig. 5c, d). The genes related to defence against stress (JAR1, CAMTA5, CBL9, HAB1), the cell wall (MIOX5, EXLB1), polyamine biosynthesis (SPDS3), photoprotection (FIB1A) and starch degradation (BAM1) were up-regulated (Table 3; Additional file 1). Conversely, the photosynthesis-related genes (PSAG, PSAO, PORA, CYP38) and a phosphatase PP2C related to ABA signalling (HAI3) were significantly repressed. GO analysis of these DEGs in A25 (Additional file 2, Fig. S3) displayed similar significative categories to control conditions uncovering 31 genes differentially expressed related to the category “response to stress”. Regarding KEGG pathways (Additional file 1, Tables S7-S8), the categories “Arginine and proline metabolism”, “Protein processing in endoplasmic reticulum” were enriched in the specific upregulated genes in A25 after salt stress.

**Table 3:** Summary of the common differentially expressed genes after 14DAT in the comparison NaCl/Control in the accession A25. It is represented both the up (FC > 1) and down-regulated genes (FC < 1), as well as the fold change (FC) and the adjusted *P*-value obtained for each gene (significant differences were considered when *P* < 0.05)

Full name	Short name	FC	*P*-value	*C. annuum* code	*A. thaliana* code
Expansin-like B1	EXLB1	6.1	4.10E-02	CA01g06350	AT4G17030
Calmodulin-binding transcription activator 5	CAMTA5	4.3	7.05E-03	CA01g14110	AT4G16150
Beta-amylase 1	BAM1	2.7	0.05	CA03g02770	AT3G23920
Calcineurin B-like protein 9	CBL9	2.7	2.25E-03	CA01g33680	AT5G47100
Hypersensitive to ABA1	HAB1	2.3	0.02	CA08g03850	AT1G72770
Jasmonate resistant 1	JAR1	2.1	0.01	CA08g08190	AT2G46370
Myo-inositol oxygenase 5	MIOX5	2.0	0.04	CA12g20180	AT5G56640
Fibrillin 1A	FIB1A	1.7	0.04	CA02g18750	AT4G04020
Spermidine synthase 3	SPDS3	1.6	0.05	CA03g19440	AT5G53120
Highly ABA-induced PP2C protein 3	HAI3	0.7	0.04	CA06g24830	AT2G29380
Cyclophilin 38	CYP38	0.5	0.05	CA02g29500	AT3G01480
Photosystem I subunit G	PSAG	0.5	0.02	CA07g20940	AT1G55670
Photosystem I subunit O	PSAO	0.4	0.04	CA06g22830	AT1G08380
Protochlorophyllide oxidoreductase A	PORA	0.1	0.04	CA10g00480	AT5G54190

For the A6 accession, 33 and 25 specific up- and down-regulated genes were respectively found after salt stress (Fig. 5c, d), in which the genes related to cell expansion (EXPA4), photosynthesis (PSBP-1, TROL, PSAE-2) and starch degradation (BAM5) were down-regulated (Table 4; Additional file 1). GO enriched categories of DEGs in A6 after salt stress (Additional file 2, Fig. S4) were similar to A25 in the same conditions but the number of genes were significatively lower. On the other hand, we highlight “Photosynthesis” as a KEGG enriched pathway in specific downregulated DEGs in the comparison A6 in control conditions and A6 after salt stress.

**Table 4:** Summary of the common differentially expressed genes after 14DAT in the comparison NaCl/Control in the accession A6. It is represented both the up (FC > 1) and down-regulated genes (FC < 1), as well as the fold change (FC) and the adjusted *P*-value obtained for each gene (significant differences were considered when *P* < 0.05)

Full name	Short name	FC	*P*-value	*C. annuum* code	*A. thaliana* code
Cellulose synthase-like D3	CSLD3	3.9	6.06E-03	CA01g07920	AT3G03050
Expansin A4	EXPA4	0.7	0.04	CA02g18410	AT2G39700
Beta-amylase 5	BAM5	0.7	0.03	CA07g12420	AT4G15210
Photosystem II subunit P-1	PSBP-1	0.7	0.04	CA07g07930	AT1G06680
Thylakoid rhodanese-like protein	TROL	0.5	0.04	CA08g08250	AT4G01050
Photosystem I subunit E-2	PSAE-2	0.5	0.04	CA06g28140	AT2G20260
Beta-amylase 5	BAM5	0.5	6.63E-03	CA07g12430	AT4G15210

Finally, we also analysed the A25 transcriptome compared to A6 under salt stress conditions. The comparison revealed 53 up- and 40 down-regulated genes, which were specifically expressed under the salt stress conditions (Fig. 5a, b). The genes related to chaperones (J8, TTA1), photosynthesis (CcdA), ion homeostasis (OCT4, PHT1;4; TIL), cell expansion (EXPA4), flavonoid biosynthesis (TT4) and ABA signalling (SnrK2.5) were up-regulated, while the genes involved in photosynthesis (PPD1, PORA) and wax biosynthesis (CER1) were down-regulated (Table 5; Additional file 1).

**Table 5:** Summary of the specific differentially expressed genes after 14DAT in the comparison A25/A6 in plants subjected to salt stress conditions. It is represented both the up (FC > 1) and down-regulated genes (FC < 1), as well as the fold change (FC) and the adjusted *P*-value obtained for each gene (significant differences were considered when *P* < 0.05)

Full name	Short name	FC	*P*-value	*C. annuum* code	*A. thaliana* code
Temperature-induced lipocalin	TIL	3.39	1.01E-03	CA07g02210	AT5G58070
Chaperone DnaJ-domain superfamily protein	J8	2.7	3.92E-03	CA00g87730	AT1G80920
Chalcone and stilbene synthase family protein	TT4	2.4	0.03	CA05g17040	AT5G13930
Organic cation/carnitine transporter4	OCT4	2.18	5.81E-03	CA07g18590	AT3G20660
Temperature-induced lipocalin	TIL	1.87	0.03	CA09g18430	AT5G58070
Class I heat shock protein, putative (DUF1423)/ Titania 1	TTA1	1.7	7.81E-03	CA04g04530	AT1G14740
SNF1-related protein kinase 2.5	SnRK2.5	1.5	0.04	CA12g16870	AT5G63650
Expansin A4	EXPA4	1.42	0.02	CA02g18410	AT2G39700
Cytochrome c biogenesis protein family	CcdA	1.42	0.04	CA07g18200	AT5G54290
Phosphate transporter 1;4	PHT1;4	1.4	0.04	CA03g05830	AT2G38940
Photosystem II reaction center PsbP family protein	PPD1	0.69	0.04	CA01g31620	AT4G15510
Eceriferum 1	CER1	0.69	0.01	CA00g87940	AT1G02205
Protochlorophyllide oxidoreductase A	PORA	0.15	0.02	CA10g00480	AT5G54190

## Discussion

In this work, we analysed the gene expression of two pepper accessions under control and salt stress conditions, called A25 and A6, previously classified as tolerant and sensitive to salt stress conditions from an agronomical and physiological point of view [12, 24]. In the present study, A25 accession exhibited under control conditions activation of genes related to cell growth and division, as well as cell wall expansion. This evidence, together with the inactivation of starch degradation and defence pathways, provides to A25 an advantage respect to A6 since we observed enhanced biomass. Indeed, several authors have already visualized differences in accessions or varieties of *Arabidopsis thaliana*, pepper or tomato [13, 24, 25] under control conditions that may influence the grade of tolerance to salt stress.

On the other side, we detected different strategies to face salt stress in the two studied accessions, what conferred at 14DAT contrasting grades of tolerance. In the several sections below, we have explained the main processes affected by this complex gene regulation network in response to salinity stress.

### Hormonal Signalling

Hormone signalling and biosynthesis have been considered an essential point of the regulation of plant tolerance or susceptibility to stress [25]. Accordingly, our results uncover several genes involved in jasmonates (JAs) and abscisic acid (ABA) synthesis, degradation or signalling that could explain the behaviour of the analysed accessions.

Jasmonates are key elements in the regulation of a wide range of processes when different abiotic stresses are present [26–28]. However, they need to be conjugated with a series of compounds to be active [29]. We found that gene jasmonate resistant 1 (JAR1), responsible of the creation of an active jasmonyl-isoleucine (JA-Ile) conjugate, was up-regulated when salt stress and control were compared in the A25 accession, but it was absent in A6. Several authors have demonstrated by external applications that JAs improve the activity of different antioxidant enzymes, growth and development, photosynthetic activity and Na^+^ homeostasis [26, 30, 31].

ABA is a well-known hormone that plays a central role in tolerance to different abiotic stresses as it performs a wide variety of functions in plant growth and development, it regulates plant water balance by stomata opening, and it plays a crucial role in osmotic stress tolerance [32]. Increasing ABA concentration and signalling are wide responses of the tolerance described by several authors, which favours stomata closure and, thus, avoids excess transpiration. However, this fact also compromises plant growth as it diminishes photosynthetic activity [25, 33]. In our experiment, we found several DEGs in A25 described as regulators of ABA, or are regulated by ABA signalling (HAB1, ERD4, CAMTA5, Tiny2, CBL9, Snrk2.5, Snrk2.10, HAI3) and, thus, play a central role in controlling tolerance.

Of all the ABA-related DEGs found in A25, one of the most relevant was the up-regulated gene hypersensitive to ABA1 (HAB1). HAB1 encodes a functional type 2C protein phosphatase (PP2C) and has been reported as a positive or negative regulator of ABA signalling, depending on the splice variant [34, 35]. Overexpression of this gene has been reported, in fact, that leads to a minor or major ABA sensitivity, modifying stomata opening and gene expression [35, 36].

A family of transcription factors, which has been reported to be regulated by ABA and plays an important role in stress tolerance, is the Calmodulin-binding transcription activators family (CAMTA) [37]. It has been demonstrated that the CAMTA family can bind to the promoters of different members of the dehydration-responsive-element-binding (DREB) transcription factors family and modulate the stress response [38]. In our case, we found the up-regulation of CAMTA5 genes and DREB member TINY2 in the A25 accession, which may indicate that both genes enhanced the response to salt stress by improving growth, development, the expression of stress-responsive genes or ABA-mediated stomatal closure [39–42].

### Biomass and cell growth

Salt stress negatively affects cell growth and plant biomass. However, greater biomass conservation is considered a sign of tolerance [43–46]. In this study, at 14DAT a better maintenance of root and aerial biomass was found in A25 compared to the A6 accession under the salt stress conditions. Biomass preservation is usually associated with the differential expression of a wide variety of genes related to cell growth and division, some of which were identified in this experiment. One of these genes is an ABA-related gene called ERD4 (early-responsive to dehydration 4), which was up-regulated in the A25/A6 comparison under both the control and salt stress conditions. This gene has been described in the bibliography as being overexpressed in tolerant transgenic *A. thaliana* plants when salt is added [47].

One of the keys to improve plant growth is defined by the ability of plants to maintain water status. After salt addition, plants undergo a reduction in the content of water in cells; in order to avoid it, plants set different mechanisms. Among them, the accumulation of a wide range of compatible osmolytes, such as proline, is crucial to help lower the Ψ_s_ [48]. Herein we demonstrated that the tolerant accession maintained at lower levels the osmotic potential and accumulated more proline content in leaves under salt stress, what can be related to the preservation of water status of plants, as has been previously reported [49]. Indeed, we detected as well a positive correlation among the up-regulation of the gene CaP5CS and the proline content (R^2^ = 0.85), what has been described as a signal of tolerance [4]. Despite these results, we do not discard the possibility that other compounds described in bibliography, such as sugars, glycinebetaine or non-compatible osmolytes such as ions were all participating to reduce the osmotic potential [48].

We also detected up-regulation of a series of genes in the A25 accession related to cell division and expansion. One of these genes was the cell division control 2 (CDC2), which regulates the G1/S and G2/M transitions in mitosis [50]. It has been demonstrated that abiotic stresses, such as drought, can negatively affect CDC2 activity [51]. As the expression in the A25 accession was 6.91-fold higher in salt stress, cell division rhythm improved. Additionally, we found an increased expression of the expansin genes in the A25 accession under salt stress compared to the control or the A6 accession, responsible for the non-enzymatically loosening and extension of plant cell walls [52]. This finding suggests that A25 improved cell wall expansion and turgor, which may lead to better growth and development, as other authors have already demonstrated [53, 54].

### Starch degradation

Abiotic stresses may also affect starch accumulation and degradation, as it may be remobilised to release energy, sugars, carbon and derived metabolites when photosynthesis is limited [55]. In general terms, under salt stress conditions a decrease of the starch content has been described, although an improved accumulation has been observed in tolerant plants [56], as we have noticed in this experiment in A25. However, a better starch degradation into soluble sugars has been also linked with tolerance to stress, since they may interact with hormones, genes and proteins, regulating diverse pathways as well as growth and development [57]. In line with this, β-amylase 1 (BAM1) was found to be up-regulated only under the salt stress conditions in the A25 accession, what would respond to transitory starch degradation in guard and mesophyll cells of mature leaves, as other authors has already observed under osmotic and salt stress conditions [58, 59].

### Ion homeostasis

When plants come into contact with salt, it is crucial to maintain ion homeostasis to avoid toxic accumulation. Plants cope with this situation by different mechanisms that can contribute to salt tolerance, some of which are very well documented in the bibliography [9, 60].

One of the most important and abundant cations in plants cells is K^+^, which decreases under salt stress conditions because of replacement with Na^+^. The enhanced K^+^ homeostasis in the A25 accession in both organs indicated that K^+^ played an pivotal role to contribute to the salt stress tolerance, as other authors have already demonstrated [9, 16]. In this line, it has been previously described that this cation, together with other compatible osmolytes, can contribute to the decrease in osmotic potential of plants [61, 62], what could indicate similar functions herein since the accumulation was more evident in A25 accession. In this experiment, additionally, we have detected that A25 may possess some mechanism to keep K^+^ inside cells by the evaluation of the DEGs; one possible candidate that could explain it is AKT1, a passive transporter that specifically introduces K^+^ into root and mesophyll cells [63, 64]. Thus we detected the up-regulation of the negative regulator of ABA signalling CBL9 (calcineurin B-like protein 9) and the down-regulation of positive regulator HAI3 (Highly ABA-Induced 3) in the A25 accession under salt stress conditions [65]. These genes play opposite roles in the regulation of AKT1 as CBL9 is a positive regulator [66], and HAI3 could be a repressor as this gene presents a high homology to HAI2 [67, 68].

The accumulation of Cl^−^ ions and especially Na^+^ in pepper plant tissues, performs diverse physiological functions [15, 16]. When Na^+^ reaches toxic levels, plants may decrease the influx into cells and improve efflux and compartmentalisation in other organelles where ions are not toxic [7]. In our experiment, we found that Na^+^ was accumulated in the roots of both accessions after 14DAT. As this accumulation was especially pronounced in A25, and root biomass had improved compared to the A6 seedlings, this effect could be associated with compartmentalisation in vacuoles or other organelles, as other authors have already demonstrated [4, 69]. Despite the negative effect on plant growth consequence of its toxic effect, accumulation of ions under salinity can help to maintain the turgor pressure of plants [15, 70]. The adjustment of the osmotic potential through inorganic ion uptake implies a much lower energy cost than that conferred by the organic molecules synthesised in cells [71].

In leaves, Na^+^ was equally accumulated in both accessions, but biomass improved only in A25, a cue that Na^+^ management was diverse in both pepper accessions. In line with this, we noticed that ion transport in leaves was closely linked to the protection of chloroplasts in A25 as we found some related genes. One of these genes was BASS1 (bile acid/sodium symporter 1), which was up-regulated in the control and salt stress treatments in the A25/A6 comparison. This gene, which encodes a symporter of Na^+^ and pantoate, a precursor of Vitamin B5, could play a double role in A25: on the one hand, it conferred protection from Na^+^ toxicity in chloroplasts to conserve photosynthesis responses; on the other hand, the pantothenate cycle was promoted [72, 73]. We also found the gene TIL (temperature-induced lipocalin), which was up-regulated in A25 compared to A6 under salinity stress, which can avoid excess Na^+^ and Cl^−^ accumulation in chloroplasts by protecting chlorophyll b degradation in this way [74].

An up-regulated gene found in the A25/A6 comparison under the salt stress conditions was OCT4 (organic cation/carnitine transporter 4), which decreases the concentration of toxic Na^+^ in the cytoplasm by accumulating in vacuoles. This family of genes is responsible for the symport of Na^+^ and organic molecules like carnitine [75, 76]. So it would play an important role in osmotic balance through ionic homeostasis in our tolerant accession.

### Photoprotection

When plants come into contact with salt stress, one of the primary affected processes is photosynthesis. The photosynthetic parameters herein analysed reflected that only A25 maintained them at 7DAT compared to the control conditions, although both accessions were equally affected at the end of the experiment. These reasons suggest that A25 kept the plant’s photosynthetic capacity levels high for longer times [77]. In addition, some genes involved in the protection of photosynthesis were differentially expressed in both accessions. In line with this, we found the up-regulation of ABA-related gene fibrillin 1A (FIB1A) in the A25 NaCl/control, which suggests that fibrillin was accumulated in chloroplasts and, consequently, could improve protection and efficiency of PSII [78]. Together with FIB1A, other previously explained genes contributed to photoprotection, such as TIL, BASS1 or JAR1.

### ROS scavenging

When photosynthesis is disturbed by salt stress, a series of secondary effects is detected, such as oxidative stress, which may lead reactive oxygen species (ROS) to toxic levels [79]. The ability to reduce the quantity of all these molecules by efficient ROS-scavenging mechanisms is vital for acquiring tolerance. In our specific case, we detected that improving the accumulation of phenolic compounds was enhanced in the A25 accession under salt stress, what has been widely described to improve the antioxidant capacity of plants [80]. Additionally, the accumulation of H_2_O_2_ in plants, as in the case of A25, has been identified by several authors as a signal of ROS damage. Nonetheless, it has been proposed in the last decades to play also a role as a secondary messenger to activate plant antioxidant processes related with abiotic stress acclimation and consequently mediate adaptative responses to abiotic stress [16], so similar functions are proposed. Accumulation of other molecules in A25 tolerant accession, such as proline, could also be implied in ROS detoxification and salt protection, as other authors have already described [81]. Regarding gene expression, we have found up-regulation of the gene SPDS3 (spermidine synthase 3) in A25 under salt stress conditions, which catalyses the formation of spermidine, a polyamine that improves multiple processes in plants, such as ROS scavenging, the K^+^/Na^+^ ratio and PSII efficiency by protecting thylakoid membranes and chlorophyll content [82–84]. We also found another up-regulated gene in A25 under salt stress conditions, called sucrose non-fermenting 1-related protein kinase 2–10 (SnRK2.10), which regulates the gene expression, protein level and/or enzymatic activity of several ROS-related enzymes, and is also involved in H_2_O_2_ accumulation and ascorbate cycle regulation in *A. thaliana* [85].

## Conclusions

After analysing the physiological parameters and DEGs of both accessions, we conclude that different tolerance strategies simultaneously took place in the A25 tolerant accession after exposure to salt stress, with ABA-signalling being a pivotal point of regulation, and an important network was established between different genes and physiological traits to reveal the complex response induced by salinity. These results provide valuable results about salt stress mechanisms of an important crop like pepper. It is noteworthy that we also found several genes that probably contributed to tolerance, but their functions have not yet been discovered.

## Methods

### Plant material

Based on previous studies [12, 24], two accessions of *C. annuum* were selected depending on their grade of tolerance to salt stress: code A6 (Pasilla bajio, Mexico) was sensitive and code A25 (Numex big Jim, Nuevo Mexico) was tolerant. All the accessions used for the present study belong to the germplasm bank placed in the Institute for Conservation and Improvement of Valencian Agrodiversity “COMAV” (Universitat Politècnica de València, Valencia, Spain). Maria José Diez, director of COMAV, verify A6 and A25 with the deposition numbers BGV013994 and BGV014452 respectively.

Seeds were sown in 104-hole seed trays filled with enriched substrate for germination. When plants had 6–8 real leaves, they were placed in 5-l polyethylene pots covered with aluminium sheet (roots were previously cleaned of substrate). Pots were filled with a nutrient solution containing (in mmol L^− 1^) 12.3 NO_3_^−^, 1.02 H_2_PO_4_, 2.45 SO_4_^2−^, 3,24 Cl^−^, 5.05 K^+^, 4.23 Ca^2+^, 2.55 Mg^2+^ and micronutrients (15.8 μM Fe^2+^, 10.3 μM Mn^2+^, 4.2 μM Zn^2+^, 43.5 μM B^+^ and 1.4 μM Cu^2+^), which was artificially aerated. The electrical conductivity (EC) and pH of this nutrient solution were 1.7 dS m^− 1^ and 6.5, respectively. The nutrient solution was added daily to compensate for absorption. After 14 days of plant acclimation, salt stress was induced by the addition of NaCl 70 mM by replacing the plant pot solution to obtain an EC of 8.5 dS m^− 1^ and a pH of 6.1. The layout design was completely randomised with 10 plants per accession and treatment.

During the culture and experiment, plants were grown in a greenhouse at the Polytechnic University of Valencia (UPV, Valencia, Spain) under natural light conditions (800–1000 μmol m^− 2^ s^− 1^), with a temperature range of 18–25 °C and 50–70% relative humidity (RH).

All the parameters were measured 14 days after stress induction, except in the photosynthetic parameters, where measurements were taken after 7 days (7DAT) and 14 days (14DAT) of treatment.

### Biomass determination

Six replications per accession and treatment were harvested at 14DAT for the biomass parameters. Aerial organs and roots were separated and weighed (FW). Immediately afterwards, they were dried by placing them in an oven at 65 °C for 72 h. After this time, everything was weighed again to determine dry weight (DW).

### Gas exchange measurements

CO_2_ fixation rate (A_N_, μmol CO_2_ m^− 2^ s^− 1^), stomatal conductance (g_s_, mol H_2_O m^− 2^ s^− 1^), substomatal CO_2_ concentration (Ci, μmol CO_2_ mol ^− 1^ air) and transpiration rate (E, mmol H_2_O m^− 2^ s^− 1^) were determined with a portable LI-COR 6400 (Li-Cor Inc.) infrared gas analyser at 7DAT and 14DAT. Measurements were taken under saturating light conditions (1000 μmol quanta m^− 2^ s^− 1^), reference CO_2_ of 400 μmol CO_2_ mol^− 1^, on fully expanded leaves (3rd-4th leaf from the apex) at a cuvette temperature of 24 °C and 75% of relative humidity. Measurements were taken from 09:00 h to 12:00 h (UT + 01:00). The layout was randomised with five replications per accession and treatment.

### Ion determination

Six replications per accession and treatment of leaves and roots were collected and dried at 65 °C for 72 h at the end of the experiment (14DAT). Dried samples were ground with a mortar and used for the ionic analysis.

With Na^+^ and K^+^, samples (0.2 g for leaves, 0.1 g for roots) were incinerated in a muffle furnace for 12 h at 550 °C. Ions were extracted with 2% nitric acid in an ultrasonic bath for 30 min at 40 °C. The concentration of such ions was determined by an ICP emission spectrometry (iCAP 6000, Thermo Scientific. Cambridge, England, UK).

Regarding chloride concentration (Cl^−^), dry plant material (0.125 g of roots and leaves) was extracted with 0.1 N HNO_3_ in 10% (v/v) acetic acid and was quantified by potentiometric titration with AgNO_3_ in a chloride analyser (Sherwood, MKII 926).

### Osmotic potential

Four replications per accession and treatment were analysed. Firstly, leaves samples were frozen in liquid nitrogen and stored at − 80 °C. After that, samples were introduced in a 1.5 mL tube and centrifuged for 10 min at 9000 x *g*. Leaf sap was measured with an osmometer (Digital osmometer, Wescor, Logan, UT, United States). Osmolyte content (mmol kg ^− 1^) was converted into MPa by the Van’t Hoff equation.

### Starch content

Starch determination was analysed according to [86] with modifications, using for that four replications per genotype and treatment. Fresh samples were frozen and ground in liquid nitrogen and stored at − 80 °C. Samples (0.3 g of leaves) were mixed with heated ethanol 80% (v/v) and boiled at 85 °C for 10 min three times. After that, samples were centrifuged at 10,000 *x g*. The precipitated was resuspended in perchloric acid 35% (v/v) and left for 24 h at room temperature. After that, samples were diluted with water and then filtered. The filtrate was then mixed with anthrone acid solution and placed in boiling water for 7.5 min. After cooling the samples, absorbance was measured at 630 nm. D-glucose was used as standard.

### Total phenol content

Total phenol content was measured according to [87] with modifications. Four replications per genotype and treatment were frozen, ground in liquid nitrogen and stored at − 80 °C. Samples (0.1 g of leaves) were mixed with 1.5 mL of 80% methanol (v/v) and extracted in an ultrasound bath at room temperature for 30 min. After that, leaf extract was diluted in extraction solution (dilution 1:4). When samples were diluted, 0.7 mL of Folin–Ciocalteu solution (Sigma-Aldrich®; 1:10 dilution), and 0.7 mL of 6% (w/v) Na_2_CO_3_ were added to samples and were incubated at room temperature in dark conditions for 1 h before measuring the absorbance at 765 nm. Standard curve was determined by the content of gallic acid.

### Hydrogen peroxide content

The H_2_O_2_ content was quantified followwing [88, 89], with modifications. Four replications per genotype and treatment were frozen and ground in liquid nitrogen and then conserved at − 80 °C. Samples (0.25 g of leaves) were mixed with 2 mL of 0.1% (w/v) trichloroacetic acid (TCA) and centrifuged at 10,000 *x g* at 4 °C for 8 min. A volume of 0.4 mL of sample was diluted with 0.6 mL of 0.1% (w/v) TCA. Afterwards, 0.5 mL of 100 mM potassium phosphate buffer (pH = 7) and 2 mL of 1 M KI were added and incubated for 1 h at room temperature under dark conditions. Absorbance was measured at 390 nm. Standard curve was determined by known concentrations of H_2_O_2_.

### Proline determination and CaP5CS gene expression

Proline content was determined according to [90]. Six replications per genotype and treatment were used for its determinations. Leaves were dried at 65 °C for 72 h and then ground with a mortar. Samples (0.02 g) were mixed with 3% sulfosalicylic acid and then the homogenate was centrifuged at 12,000 x *g* for 5 min. Afterwards, glacial acetic acid and ninhydrin reagent were added to the samples. and boiled at 100 °C for 1 h. After cooling the samples, absorbance was measured at 520 nm. Proline content was calculated using a known standard curve.

Additionally, the putative gene Delta-1-pyrroline-5-carboxylate synthase of pepper (CaP5CS, CA06g06110), implied in the synthesis of proline, was measured in leaves at 14DAT following the methodology described below in the section “Validation of Microarrays Analysis by Real-Time Quantitative PCR (RT-qPCR)”. The primers used for its determination were (5′-3′): TTTAGTGATGGGTTCCGCTTTG (Forward) and CAATCCCTCGAVCTCCAACTC (Reverse).

### Extraction and quality measurement of Total RNA

Three replications of leaves per treatment and accession were frozen in liquid nitrogen immediately after harvest and conserved at − 80 °C at 14DAT. At the time of RNA extraction, samples were ground to a fine powder with a mortar and liquid nitrogen. Total RNA was extracted using the MACHEREY-NAGEL NucleoSpin® RNA kit. Approximately 0.1 g was weighed, and RNA was obtained following the protocol “RNA purification from cultured cells and tissue” by the producer; DNase treatment was used to remove DNA from samples and was acquired from the same producer. Total RNA was eluted in 50 μL of RNAse-free water and was immediately aliquoted and conserved at − 80 °C. The total RNA samples with 260/280 and 260/230 ratios > 2 (measured by a NanoDrop ND1000) and RNA integrity (RIN) value > 7.0 (measured by the Agilent 5067–1511 Bioanalyzer 2100 System) were used for microarray hybridisation.

### Microarray hybridisation

The RNA extracted from the leaf samples was prepared for microarray hybridisation at the Genomic Service of the IBMCP Institute (Instituto de Biología Molecular y Celular de Plantas) in Valencia (Spain) by Agilent technologies. cDNA synthesis and labelling on Agilent Tomato microarrays were carried out using the Agilent One Colour RNA Spike-in Kit and the Agilent Low Input Quick Amp Labeling Kit. Microarray hybridisation and washing were next performed with the Agilent Gene Expression Hybridization kit and Gene Expression Wash Buffers. Agilent microarray 4*44 k (Agilent G2519F) was selected for hybridisation (reference AMADID 22270 *Tomato*). Microarray scanning was done with a GenePix 4000B (Axon Molecular Devices, Sunnyvale, USA) and data were extracted by the Agilent Feature Extraction software, version 9.5.1.

### Microarray data analysis

The obtained spot intensity values were analysed on the Babelomics 5 platform [91]. Firstly, raw data were normalised, which consisted in a background correction, rescaling all the microarrays to a unique final distribution and reshaping data to a suitable distribution. At this point, data were transformed from tomato probes to pepper and *Arabidopsis thaliana* genes by the Bioinformatics service at the IBMCP Institute in Valencia (Spain) to then take the average among all the probes of the same pepper gene. Raw data were then separated into categories (accession and treatment) and analysed by a class comparison test. All the differentially expressed genes (DEGs) of the class comparison, both up- and down-regulated, were described as their orthologue of *A. thaliana* by the database of Araport 11. Using the resultant DEGs, GO classification has been developed using the platform Bio-Analytic Resource for Plant Biology (http://bar.utoronto.ca/#) and classified in “Biological Process”, “Molecular Function” and “Cellular Component”. Additionally, DEGs were also subjected to a KEGG enrichment analysis, performed with DAVID Functional Annotation Tool [92].

### Validation of microarrays analysis by real-time quantitative PCR (RT-qPCR)

The RNA of leaves used for microarray analyses was used as well for validation of the obtained results from the class comparisons. For that purpose, firstly the RNA samples were retrotranscribed by the use of PrimeScript RT reagent kit (Takara Bio) in a total volume of 20 μL. After that, all the samples were fifty-fold diluted to perform RT-qPCR. A volume of 2 μL of diluted sample was used per well (total volume of 20 μL) and SYBR premix Ex Taq (Tli RNseH plus, Takara Bio) was used to conduct the reaction. Three technical replicates were evaluated per every biological replicate. Additionally, a relative standard curve was designed to obtain the mean relative expression to get the final results. RT-qPCR was carried out on a StepOnePlus Real-Time PCR System (Life Technologies), and the sequence of reactions was firstly an incubation at 95 °C for 10 min, followed by 40 cycles of 95 °C and 60 °C for 15 s and 1 min, respectively. Amplification specificity was estimated if a unique peak was found in the melting curve of each gene analysed. EF1α and β-TUB were used as reference genes, described by [93, 94], respectively, since they concluded that they were suitable for the studies of salt stress in pepper plants. Primers sequences and results obtained have been detailed in Additional file 3.

### Statistical analysis

The experiment layout was a completely randomised design. The data from the biomass, gas exchange measurements, ion concentration analyses, osmotic potential, starch, total phenols, H_2_O_2_ and proline content and gene relative expression were subjected to a two-way ANOVA (Statgraphics Centurion XVI for Windows, Statistical Graphics Corp.), where both accession and treatment were considered to be the factors of the analysis. With the photosynthesis parameters, 7DAT and 14DAT were analysed independently. As the interaction between both factors was significant, a one-way ANOVA was performed by joining both factors of the two-way ANOVA. Ulterior comparisons were made using Fisher’s least significance difference (LSD) test at *P* < 0.05 with the same software.

Class comparison analyses of the microarrays were done using the Babelomics platform. Different treatments of the same accession (Salt/Control) and different accessions of the same treatment (A25/A6) were compared by a Limma test to compare genes, and a Benjamini and Hochberg test was run to reduce the false discovery rate. The adjusted *P*-value was selected at 0.05.

## Supplementary Information

**Additional file 1: Table S1-S12.** Total differentially expressed genes when A25 and A6 accessions are compared, in both control and salt stress conditions (Table S1-S6) and when NaCl and control conditions are compared, in both A25 and A6 accessions (Table S7-S12). KEGG pathway was included when statistical significance was found.

**Additional file 2: Figure S1-S4.** GO classification of the DEGs found of the class comparisons.

**Additional file 3: Table S13, Figure S5.** Validation of Microarray analysis by RT-qPCR by a selection of DEGs.

## Data Availability

All data generated and/or analysed during this study are included in this published article and its supplementary information files.

## Abbreviations

7DAT: 7 days after treatment
14DAT: 14 days after treatment
ABA: Abscisic acid
A_N_: CO_2_ fixation rate
AKT1: Arabidopsis K^+^ transporter 1
BAM1: beta-amylase 1
BAM5: beta-amylase 5
BASS1: Sodium bile acid symporter family
CAMTA5: Calmodulin-binding transcription activator 5
CBL9: Calcineurin B-like protein 9
CCB3: Cofactor assembly, complex C (B6F)
CcdA: Cytochrome c biogenesis protein family
CDC2: Cell division control 2
CEL5: Cellulase 5
CER1: Eceriferum 1
Ci: Substomatal CO_2_ concentration
CSD1: copper/zinc superoxide dismutase 1
CSLE1: Cellulose synthase-like E1
CYP38: Cyclophilin 38
DEG: Differentially expressed gene
DW: Dry weight
E: Transpiration rate
EC: Electrical conductivity
EXLB1: Expansin-like B1
EXPA4: Expansin A4
EXPA13: Expansin A13
FIB1A: FIBRILLIN 1A
FW: Fresh weight
gs: Stomatal conductance to water vapour
HAB1: Hypersensitive to ABA1
HAI3: Highly ABA-induced PP2C protein 3
J8: Chaperone DnaJ-domain superfamily protein
JA: Jasmonate
JAR1: Jasmonate resistant 1
KASI: 3-ketoacyl-acyl carrier protein synthase I
LACS2: Long-chain acyl-CoA synthetase 2
MIOX1: Myo-inositol oxygenase 1
MIOX5: Myo-inositol oxygenase 5
NDHG: NADH, ubiquinone/plastoquinone oxidoreductase, chain 6
OCT4: Organic cation/carnitine transporter 4
PHT1;4: Phosphate transporter 1;4
PME1: Pectin methylesterase 1
PMEI13: Plant invertase/pectin methylesterase inhibitor superfamily protein
PORA: Protochlorophyllide oxidoreductase A
PP2C: Protein phosphatase 2C
PPD1: Photosystem II reaction centre PsbP family protein
PRX66: Peroxidase 66
PRX71: Peroxidase 71
PSII: Photosystem II
PSAE-2: Photosystem I subunit E-2
PSAG: Photosystem I subunit G
PSAO: Photosystem I subunit O
PSBP-1: Photosystem II subunit P-1
RH: Relative humidity
ROS: Reactive oxygen species
SnrK2.5: SNF1-related protein kinase 2.5
SnRK2.10: SNF1-related protein kinase 2.10
SPDS3: Spermidine synthase 3
TIL: Temperature-induced lipocalin
TROL: Thylakoid rhodanese-like protein
TT4: Chalcone and stilbene synthase family protein
TTA1: Class I heat shock protein, putative/Titania 1
TUB8: Tubulin beta 8
WS6D: o-acyltransferase (WSD1-like) family protein
XK-1: Xylulose kinase-1
