# Supplementary material for: Uncovering salt tolerance mechanisms in pepper plants: a physiological and transcriptomic approach
Source: BMC Plant Biol. 2021 Apr 8;21:169. doi: 10.1186/s12870-021-02938-2 (PMC8028838; doi:10.1186/s12870-021-02938-2)
Supplement: Supplementary file 2 — Additional file 2: Figure S1-S4. GO classification of the DEGs found of the class comparisons. [file 12870_2021_2938_MOESM2_ESM.docx]

**Additional File 2. GO classification of the DEGs found of the class comparisons**


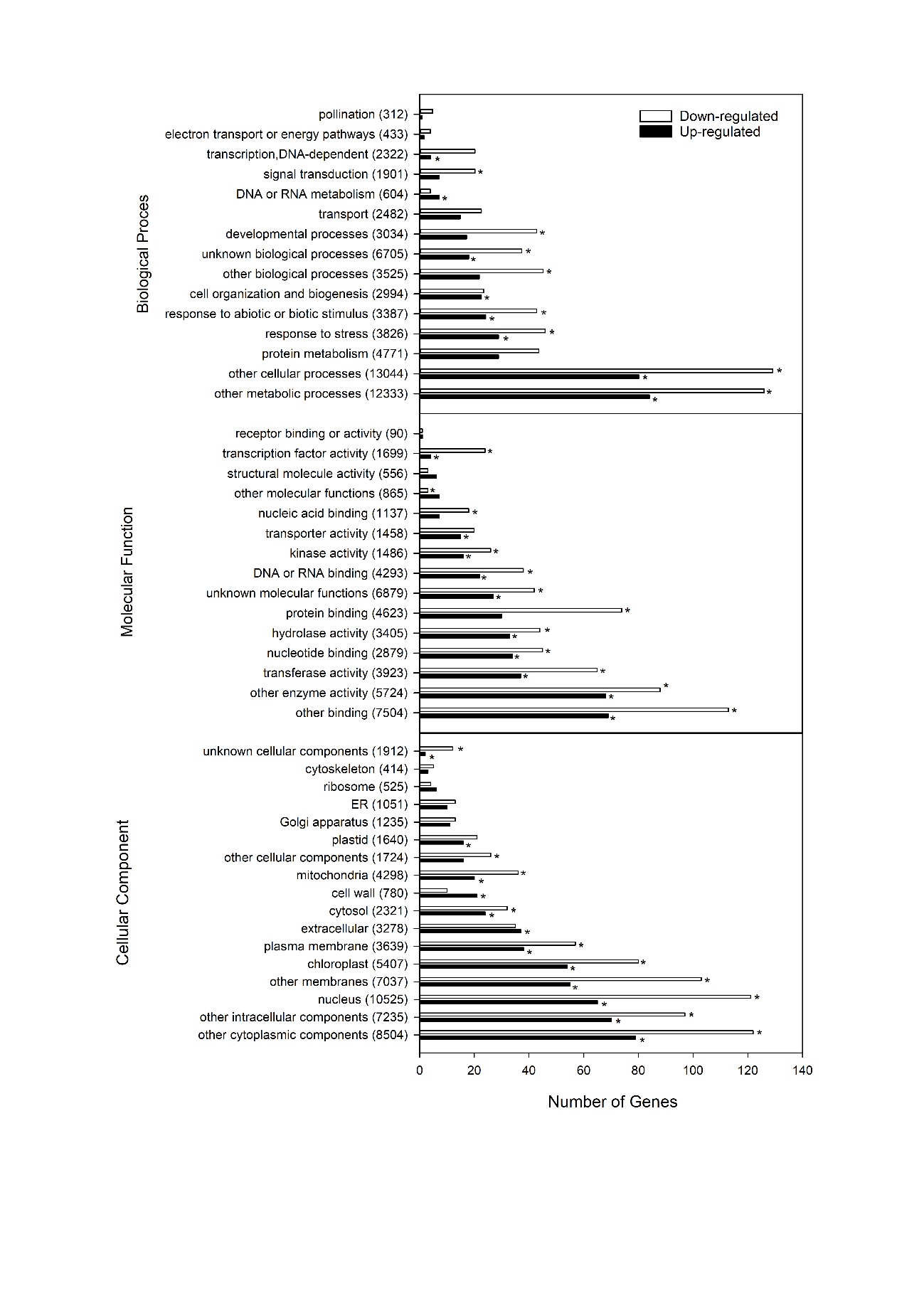


**Fig S1.** GO Classification of the DEGs found in the comparison A25 vs A6 under control conditions at 14DAT. All the up- (FC>1; black bars) and down-regulated (FC<1; white bars) genes have been represented. Asterisks are present when the *P*-value<0.05. GO classification and statistics has been performed in the platform Bio-Analytic Resource for Plant Biology (http://bar.utoronto.ca/#).


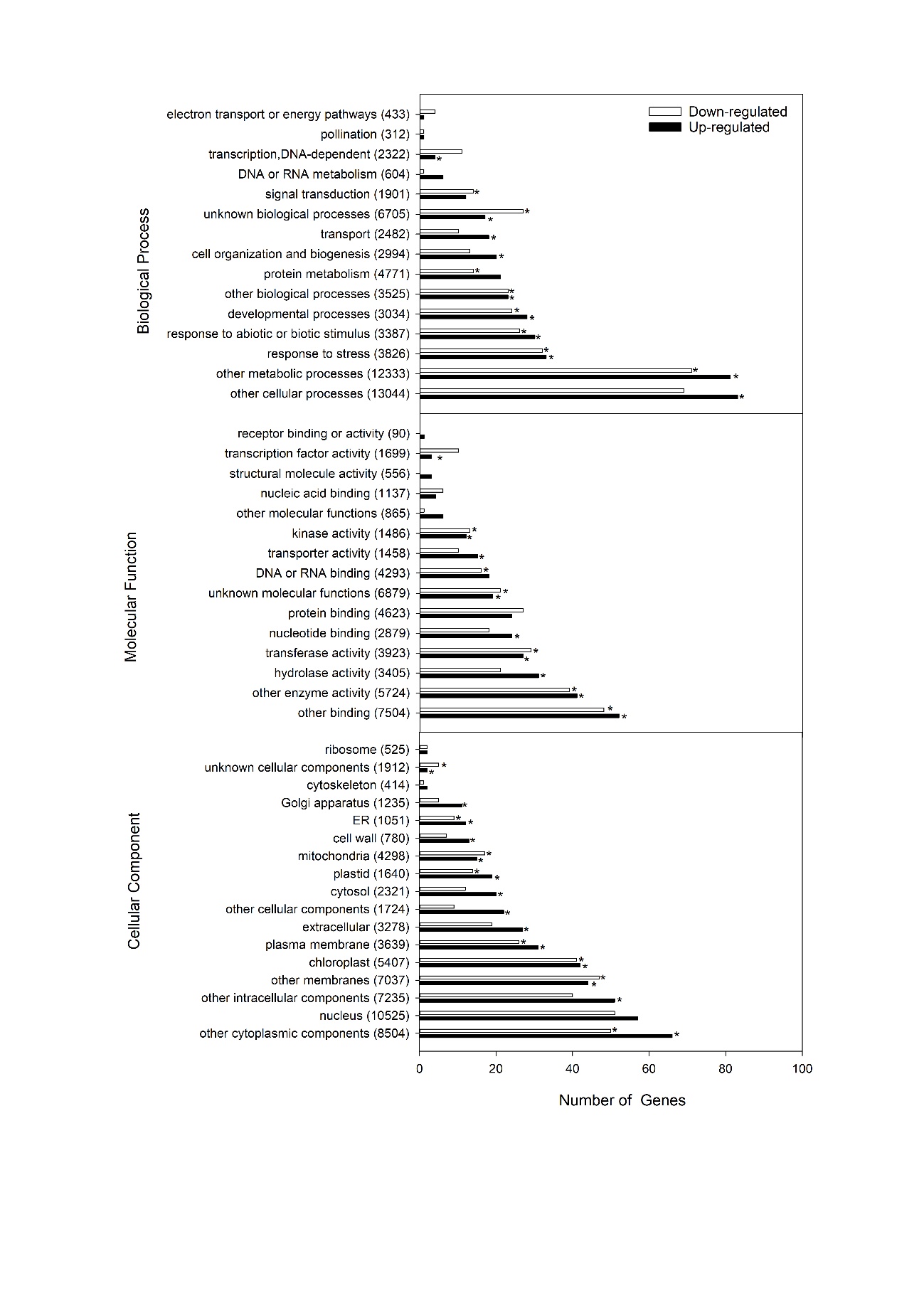


**Fig 2S.** GO Classification of the DEGs found in the comparison A25 vs A6 under salt stress conditions at 14DAT. All the up- (FC>1; black bars) and down-regulated (FC<1; white bars) genes have been represented. Asterisks are present when the *P*-value<0.05. GO classification and statistics has been performed in the platform Bio-Analytic Resource for Plant Biology (http://bar.utoronto.ca/#).


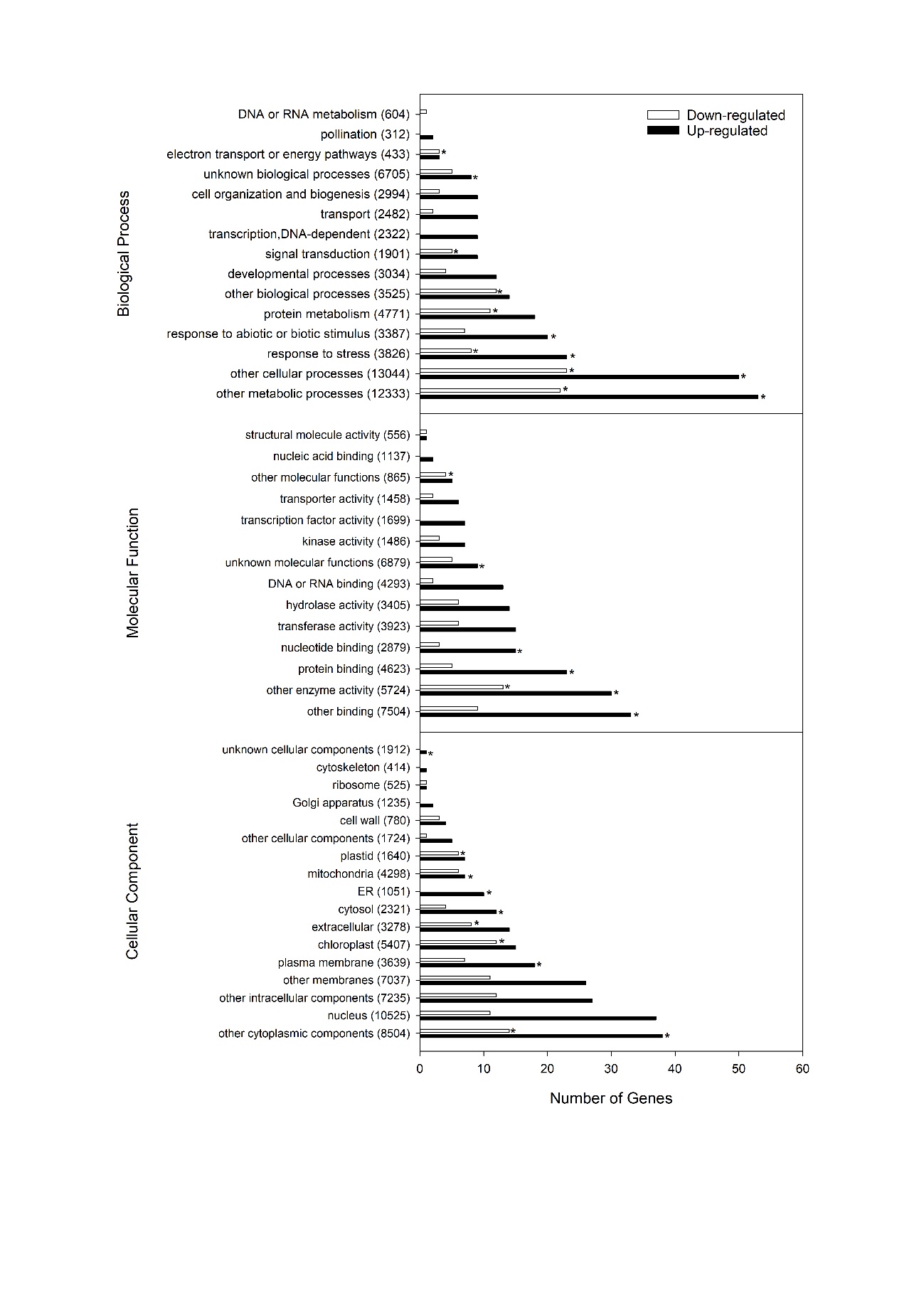


**Fig S3.** GO Classification of the DEGs found in the comparison salt stress vs control conditions in the accession A25 at 14DAT. All the up- (FC>1; black bars) and down-regulated (FC<1; white bars) genes have been represented. Asterisks are present when the *P*-value<0.05. GO classification and statistics has been performed in the platform Bio-Analytic Resource for Plant Biology (http://bar.utoronto.ca/#).


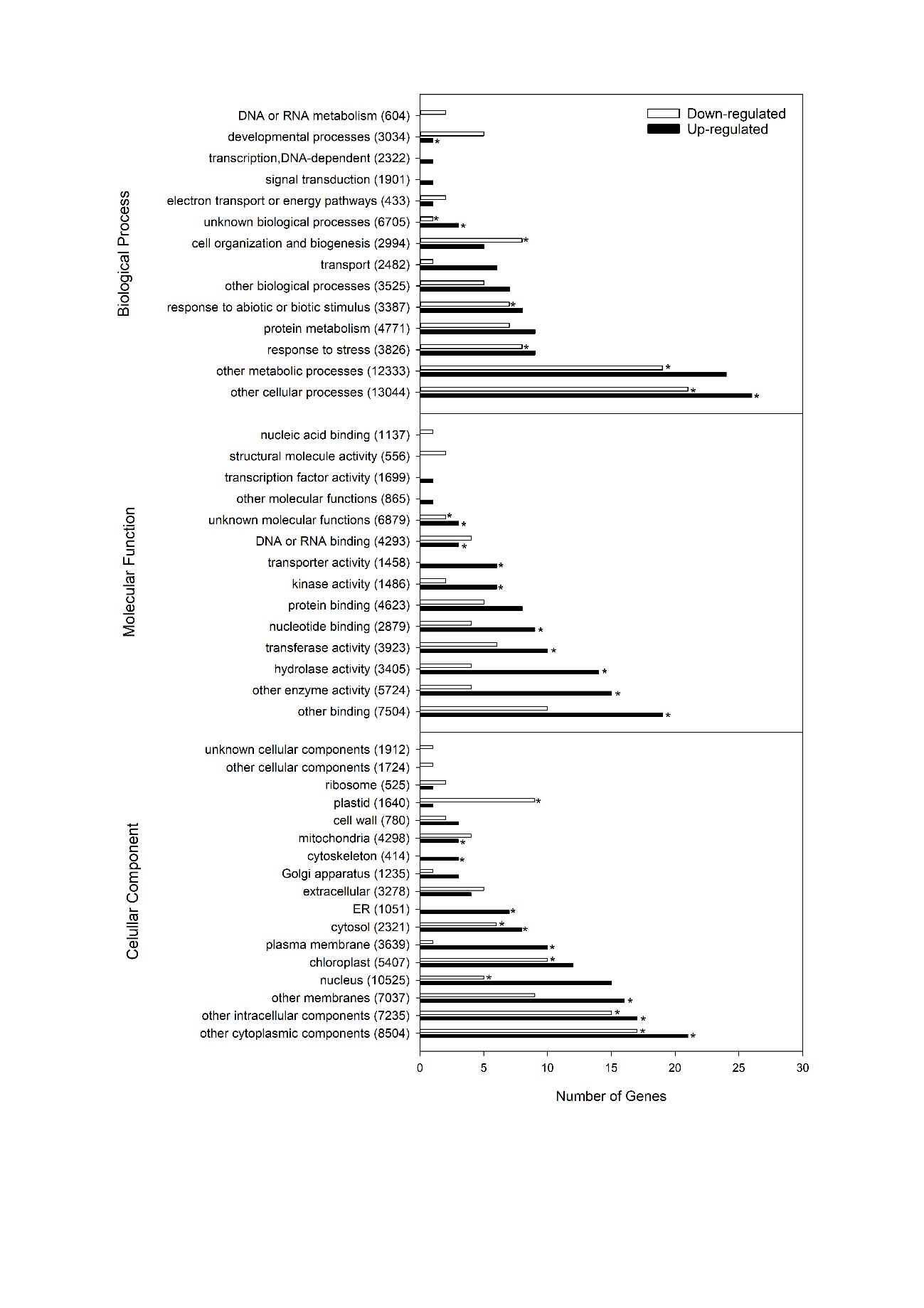


**Fig S4.** GO Classification of the DEGs found in the comparison salt stress vs control conditions in the accession A6 at 14DAT. All the up- (FC>1; black bars) and down-regulated (FC<1; white bars) genes have been represented. Asterisks are present when the *P*-value<0.05. GO classification has been performed in the platform Bio-Analytic Resource for Plant Biology (http://bar.utoronto.ca/#).
