# Supplementary material for: Uncovering salt tolerance mechanisms in pepper plants: a physiological and transcriptomic approach
Source: BMC Plant Biol. 2021 Apr 8;21:169. doi: 10.1186/s12870-021-02938-2 (PMC8028838; doi:10.1186/s12870-021-02938-2)
Supplement: Supplementary file 3 — Additional file 3: Table S13, Figure S5. Validation of Microarray analysis by RT-qPCR by a selection of DEGs. [file 12870_2021_2938_MOESM3_ESM.docx]

**Additional File 3. Validation of Microarray analysis by RT-qPCR by a selection of DEGs**

**Table S13.** Primer sequence of the selected genes for validation, both forward (FW) and reverse (RV) sequence; amplification length, and annealing temperature of the amplified product in RT-qPCR. Reference genes have been selected according to [1, 2].

| Name | *C. annuum* code | | Sequence (5'-3') | Tm | Amplification length (nt) |
| --- | --- | --- | --- | --- | --- |
| TIL | CA07g02210 | FW | TCCCTCAAGATTTCAGCCCAAAG | 57.3 | 102 |
|  |  | RV | CCACCACTCCATGTCTCATTTAACA | 57 |  |
| PORA | CA10g00480 | FW | GCAACAACAGGACTATTCAGAGAAC | 55.8 | 168 |
|  |  | RV | CCAGCTCCAGTATACACCTGAT | 55.9 |  |
| EXLB1 | CA01g06350 | FW | GCATTTGACGTTGAAGCTGTCG | 57.2 | 94 |
|  |  | RV | CTGGCATGTCCCATACTGCT | 57.3 |  |
| EF1α | AY496125 | FW | TGAAGAATGGTGATGCTGGC | 55.6 | 79 |
|  |  | RV | GGTGGGTATTCAGCAAAGGT | 55.1 |  |
| β-TUB | EF495259.1 | FW | GAGGGTGAGTGAGCAGTTC | 55.2 | 152 |
|  |  | RV | CTGTCGCATCCTGGTATTGT | 54.8 |  |


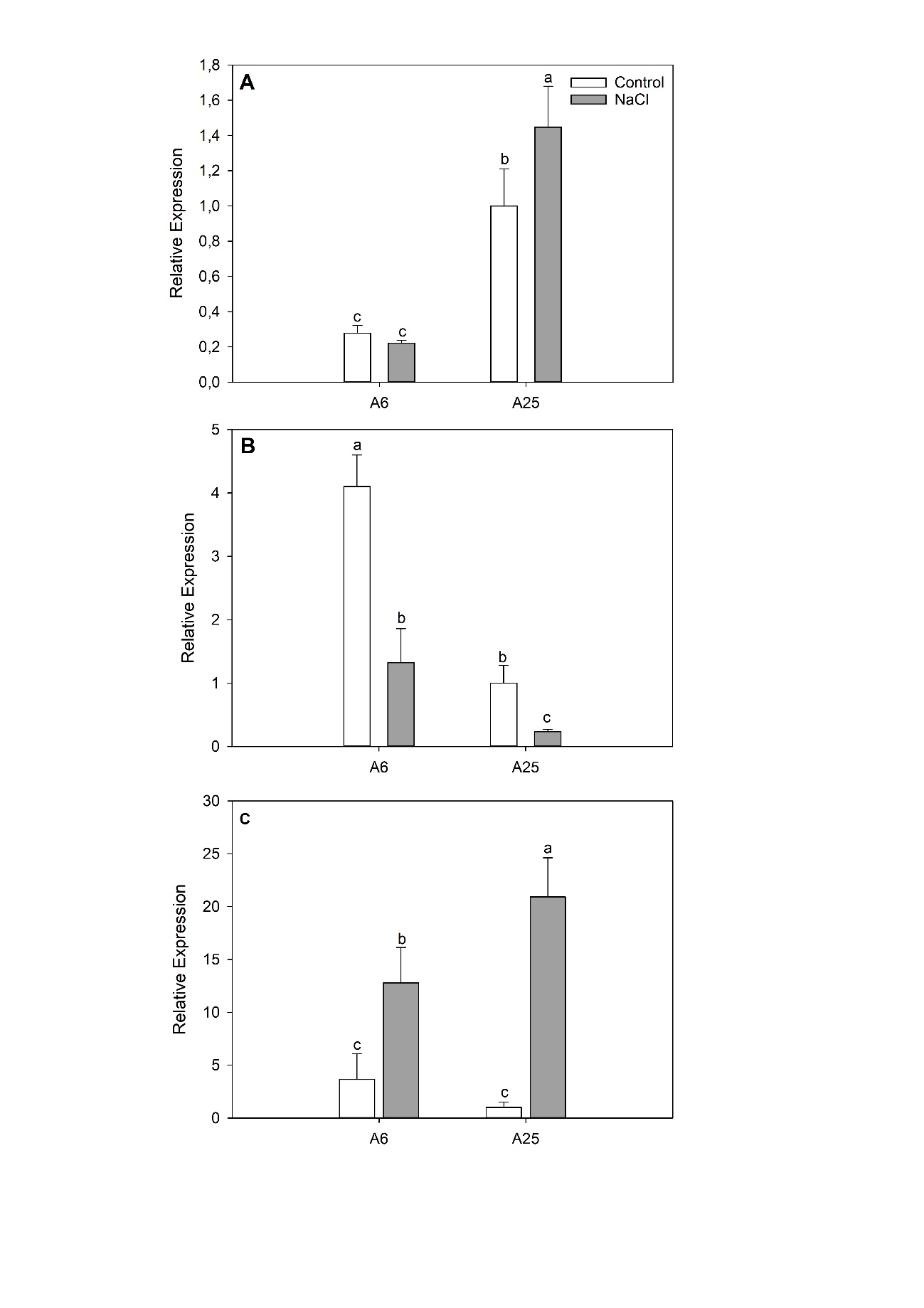


**Fig S5.** Relative expression of the genes CA07g02210 **(A)**, CA10g00480 **(B)** and CA01g06350 **(C)** for the accessions A6 and A25 subjected to control (white bars) or salt stress conditions (grey bars) at 14DAT. The error bars belong to the standard deviation. Different letters indicate significant differences at *P*<0.05 (LSD test).
